# Has socioeconomic equity increased in somatic specialist care: a register-based cohort study from Finland in 1995–2010

**DOI:** 10.1186/1472-6963-14-430

**Published:** 2014-09-24

**Authors:** Kristiina Manderbacka, Martti Arffman, Ilmo Keskimäki

**Affiliations:** Service System Research Unit, National Institute for Health and Welfare, P. O. Box 30, 00271 Helsinki, Finland; Service System Department, National Institute for Health and Welfare, P. O. Box 30, 00271 Helsinki, Finland; School of Health Sciences, 33014 University of Tampere, Tampere, Finland

**Keywords:** Specialised hospital care, Socioeconomic position, Register-based study, Health services research

## Abstract

**Background:**

Equal access to health care according to need is an important goal for health policy in Finland. Earlier research in Finland and elsewhere has mainly been cross-sectional, but the results have implied that the goal has not been fully realised in somatic specialist hospital care. This study examines trends in socioeconomic equity in use of somatic specialist hospital care.

**Methods:**

We used register data on somatic specialist hospital admissions among 25–84 year-old persons in Finland in 1995–2010 with individually linked register-based socio-demographic information. We calculated age-standardised admission rates per 100,000 person years by income, examined risk ratios using Poisson regression models and computed concentration indices separately for men and women. Linear trends in the socioeconomic distribution of admissions and surgical procedures were estimated with linear regression models for annual concentration indices.

**Results:**

Overall, use of somatic specialist hospital care decreased steadily throughout the study period. A stepwise inverse income pattern was found in hospitalisation risk and in non-surgical admissions: the lower the income group, the higher the risk. The relative admission risk was approximately two times higher in the lowest income group compared to the highest among both genders. Few differences were found in surgical admissions. Income group differences remained stable in hospitalisations and surgical admissions, but increased in non-surgical admissions during the study period. An inverse pattern of increasing operation rates with decreasing income was found in primary hip and knee replacement operations, and in lower limb amputations. A similar pattern emerged during the study period in coronary revascularisations. There were no differences were found in lumbar fusion or lumbar disc operations, prostatectomies or appendectomies. Income group differences in hysterectomies disappeared during the study period.

**Conclusions:**

While the results of the current study suggest that use of somatic specialist care declined in line with improving population health in 1995–2010, the increase of socioeconomic health differentials was only partly reflected in the distribution of somatic specialist hospital care. Further research is needed to evaluate the need to improve use and content of specialised hospital care among the low-income groups in order to improve equity in health care.

## Background

Equity in health and equal access to health care according to need are important goals for health policy in many Western countries, including Finland. The structure of the Finnish health care system, in general, supports equal access to health care. The system is mainly financed by tax revenues, while user fees are, in general, low [1]. There are, however, some supply-related barriers to equal access. While ambulatory services are primarily provided by the public sector, there have been difficulties in access. Private ambulatory services, partly refunded by the National Health Insurance, are available, especially in cities and larger municipalities, but patients’ co-payments are high. Occupational health care provides easy access to ambulatory care for employees free of charge. Together, municipalities form hospital districts which organise and provide specialist medical services for their residents. While general practitioners act as gate keepers for public specialist services in the public sector and in occupational health care, no gate keeping is exercised in private services.

Accordingly, socioeconomic differences in the use of ambulatory services have been reported to be especially large in Finland compared to other European countries [2, 3]. As for specialist hospital care, an earlier study examining 1988 and 1996 reported that in both years, in line with higher morbidity, low-income groups used somatic specialist hospital care more than the better-off [4]. However, increasing differences were found in the content of care: lower income group patients underwent fewer common surgical operations than higher income group patients. Similar differences in specialist hospital care have been reported earlier in other countries [5] and in the use of some elective surgical operations both in Finland [6] and internationally [7, 8] and in the care of specific diseases like coronary heart disease both in Finland and elsewhere [9–11]. Additionally, studies have reported socioeconomic differences in mortality amenable to health care interventions and the differences have been reported to be especially large in mortality amenable to specialist health care interventions [12]. Earlier studies have not examined long-term trends in socioeconomic differences in use of somatic specialist hospital care.

We aim to fill this gap by examining 16-year time trends in socioeconomic differences in access to somatic specialised hospital care from 1995 to 2010 using service use as a proxy. We examined trends in overall service use and surgical and non-surgical care. We further examined elective surgery, which is a useful exemplar for studying access to somatic specialist hospital care since it exhibits a strong element of discretion on the part of patient to present and on the part of health service providers as to how and when treatment is offered.

## Methods

### The data

Hospital data from 1995 to 2010 was used to examine changes in the socioeconomic distribution of somatic specialist hospital use. Data on all somatic hospital admissions were obtained from the Finnish Hospital Discharge Register covering all hospital discharges in all public and private hospitals in Finland. The hospital data were individually linked to socio-demographic data at Statistics Finland using the personal identification code unique to each resident. Each record was linked with information referring to 31 December of the preceding year. Age was classified into five-year age bands to match the age groups in population at risk tables. Family disposable income was extracted from the Employment Statistics based on tax registers and including earned (e.g. wages, benefits and pensions) and capital income subject to taxation and non-taxable social security benefits, such as child benefits, housing allowances and income support, and excluding paid taxes. We adjusted the income data for family size using the OECD equivalence scale [13]. The study population was classified into income groups according to family disposable income based on limits derived from the population at risk tables. Persons who were not permanent residents in Finland and those aged under 25 years or over 84 years at the beginning of the entry year, persons who had psychiatric hospital care only, and hospital admissions due to child-birth only were excluded from the data. Further, those in long-term care were excluded from the data, since family income could not be determined for this group reliably from the registers used.

#### Outcome measures

We examined the annual risk of hospitalisation among Finnish residents. Hospital admissions were also classified into surgical and non-surgical admissions. In additional analyses, the admissions were classified into Diagnosis Related Groups (DRG) according to the Finnish version of the Nordic DRG classification system. DRGs were further grouped into Major Diagnostic Categories (MDC). If two or more successive hospital admissions’ MDC were the same and the date of the subsequent admission was within two days from the discharge date of the preceding admission, these were considered as a single admission. Seven procedures were selected to study common and usually elective surgical procedures: coronary revascularisations, primary hip and knee replacement operations, lumbar disc operations, lumbar fusion operations, hysterectomies and prostatectomies. Lower limb amputations were included as a non-discretionary operation and appendectomies were included for comparative reasons, since appendicitis was expected not to vary by income. Coronary revascularisations include coronary artery bypass grafting (CABG) as well as coronary angioplasties (PCA). Hip replacements performed in the context of a femur fracture (ICD 10 code S72, ICD 9 codes 820–821) were excluded from the analyses. Surgical procedures were coded according to the classification of procedures of the Finnish Hospital League in 1995 [14] and thereafter according to the NOMESCO classification [15].

The study protocol was approved by the Ethics Committee of the National Institute for Health and Welfare (decision number 7/2011 §362-375). Permission to use the above described register data was obtained from the National Institute for Health and Welfare as well as Statistics Finland.

#### Statistical methods

Age-adjusted rates per 100,000 person years were calculated by income quintile using direct standardisation for men and women separately for each of the study years with the standard population being Finnish residents in 2010. Risk ratios for each income group were calculated separately for men and women for each of the study years using Poisson regression models and adjusting for age. Further, concentration indices were computed using the approach presented by Kakwani et al. [16] and Doorslaer et al. [17] separately for men and women and adjusted for age. The number of income classes used in the computation of the indices was 20 instead of 5. The concentration index (C) gives summary information about the whole distribution of the studied phenomenon in a single value. The index is restricted to values between -1 and 1. Negative values indicate that those with low incomes are hospitalised or undergo surgical operations relatively more often than those with high incomes and vice versa. A value of 0 indicates that the distribution is income neutral. Changes in the socioeconomic distribution of admissions and surgical procedures were analysed by estimating linear trends from linear regression models for annual Cs. Uncertainty in the Cs was taken into account by using the inverse of the standard errors as weights. Statistical analyses were conducted using the SAS system for Windows, release 9.3.

## Results

There were between 187,960 and 201,709 hospitalisations among men and between 218,146 and 248,403 among women annually during the study period. Overall, the use of somatic specialist hospital care decreased steadily throughout the study period (Table 1). This was especially the case with non-surgical admissions, the rate of which dropped to almost half during the study period among both genders. The rate for surgical admissions remained relatively stable between 1995 and 2010. Nevertheless, non-surgical admissions were more common than surgical admissions throughout the study period.

**Table 1:** **Socioeconomic differences in specialised hospital care by income in Finland in 1995 - 2010**

	Year	Lowest	2	3	4	Highest	All	Concentration index
		10 ^3^ 95% CI	10 ^3^ 95% CI	10 ^3^ 95% CI	10 ^3^ 95% CI	10 ^3^ 95% CI	10 ^3^ 95% CI	
Men								
Hospitalisation	1995	161 (159,163)	151 (149,152)	145 (144,147)	128 (126,129)	108 (107,110)	136 (136,137)	-0.073 (-0.139,-0.007)
	2000	164 (162,166)	146 (145,148)	132 (131,133)	116 (115,118)	102 (100,103)	129 (129,130)	-0.083 (-0.142,-0.025)
	2005	144 (142,146)	141 (140,143)	133 (131,134)	116 (114,117)	108 (107,110)	127 (126,127)	-0.056 (-0.110,-0.003)
	2010	131 (130,133)	129 (127,130)	114 (112,115)	109 (108,110)	94 (93,95)	114 (113,114)	-0.060 (-0.100,-0.019)
Non-surgical	1995	179 (177,181)	167 (166,169)	154 (152,155)	126 (125,128)	101 (99,102)	143 (142,143)	-0.108 (-0.180,-0.037)
admissions	2000	179 (177,181)	151 (150,153)	125 (123,126)	103 (101,104)	85 (83,86)	125 (124,125)	-0.135 (-0.207,-0.063)
	2005	140 (138,142)	128 (127,130)	108 (107,110)	88 (87,90)	76 (75,77)	105 (105,106)	-0.120 (-0.181,-0.058)
	2010	117 (115,118)	102 (101,103)	81 (80,82)	69 (68,70)	52 (51,53)	81 (81,82)	-0.145 (-0.194,-0.096)
Surgical	1995	88 (86,90)	87 (86,88)	88 (86,89)	80 (79,81)	72 (70,73)	82 (82,83)	-0.035 (-0.104,0.034)
admissions	2000	86 (85,88)	86 (84,87)	82 (81,83)	75 (74,76)	69 (67,70)	79 (78,79)	-0.037 (-0.097,0.024)
	2005	83 (83,85)	91 (90,93)	93 (92,94)	83 (82,84)	80 (78,81)	86 (85,86)	-0.011 (-0.073,0.050)
	2010	82 (81,83)	89 (88,90)	84 (83,85)	85 (84,86)	77 (76,78)	83 (83,84)	-0.008 (-0.059,0.043)
Women								
Hospitalisation	1995	164 (162,165)	156 (154,157)	147 (146,148)	132 (131,134)	115 (113,116)	142 (142,143)	-0.069 (-0.123,-0.016)
	2000	172 (170,173)	147 (146,148)	138 (137,139)	123 (121,124)	109 (108,110)	137 (136,137)	-0.081 (-0.127,-0.035)
	2005	147 (146,149)	139 (138,140)	133 (131,134)	117 (115,118)	111 (110,113)	129 (129,130)	-0.057 (-0.096,-0.017)
	2010	133 (131,134)	125 (124,126)	111 (110,112)	109 (107,110)	96 (94,97)	114 (113,114)	-0.059 (-0.103,-0.014)
Non-surgical	1995	148 (147,150)	134 (133,135)	116 (115,118)	98 (97,99)	81 (79,82)	115 (114,115)	-0.120 (-0.182,-0.057)
admissions	2000	143 (142,145)	111 (110,112)	94 (93,95)	76 (75,77)	66 (64,67)	97 (97,97)	-0.149 (-0.195,-0.103)
	2005	112 (111,113)	93 (92,94)	80 (79,81)	65 (64,66)	58 (57,60)	88 (80,81)	-0.132 (-0.175,-0.088)
	2010	93 (92,94)	75 (75,76)	59 (58,60)	53 (52,53)	41 (40,42)	63 (63,64)	-0.151 (-0.200,-0.102)
Surgical	1995	105 (103,106)	104 (103,105)	103 (102,104)	96 (95,97)	86 (85,87)	98 (98,99)	-0.039 (-0.093,0.015)
admissions	2000	112 (111,114)	104 (103,105)	102 (101,103)	94 (93,95)	85 (84,86)	99 (98,99)	-0.047 (-0.098,0.003)
	2005	103 (101,104)	105 (104,106)	104 (103,105)	93 (92,94)	90 (89,92)	99 (99,100)	-0.028 (-0.075,0.020)
	2010	96 (95,97)	98 (97,99)	90 (89,91)	91 (90,92)	82 (81,83)	91 (91,92)	-0.025 (-0.076,0.025)

Trends among men and women aged 25–84 years in Finland between 1995 and 2010 (Age-standardised rates per 1000 for hospitalisations and for surgical and non-surgical admissions and their 95% confidence intervals).

**Figure 1:**
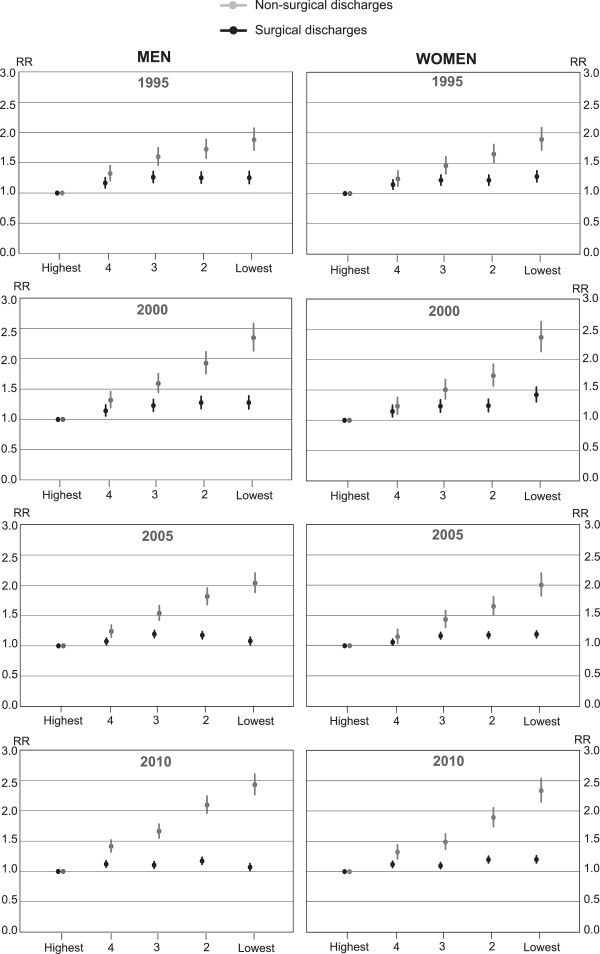
**Surgical and non-surgical admissions in specialised hospitals by income in Finland in 1995–2010.** Risk ratios for surgical and non-surgical admissions in specialised somatic hospitals among men and women aged 25–84 years by income quintile in Finland between 1995 and 2010 (Age-standardised risk ratios and their 95% confidence intervals).

A stepwise inverse income pattern was detected in annual hospitalisation rates throughout the study period: the higher the income, the lower the hospitalisation rate (Table 1). However, the difference between the two lowest income quintiles was small, especially among men. A congruent pattern appeared in rates for non-surgical admissions throughout the study period. The relative risk of admission was approximately two times higher in the lowest income group compared to the highest among both genders. Relative differences in non-surgical admissions increased during the study period (Figure 1). While rates for surgical admissions decreased modestly in the lowest income quintile and increased in the highest quintile among men, the differences between income groups measured by concentration index were statistically non-significant among both men and women in nearly all of the study years. While the relative operation risk was larger in all other income quintiles compared to those in the highest among men especially toward the study period, the risk ratios did not differ statistically significantly from each other. A linear trend of increasing differences was detected in concentration indices for non-surgical admissions among both men (p = 0.019) and women (p = 0.006). The concentration indices remained stable in hospitalisations and in surgical admissions among both genders.

**Figure 2:**
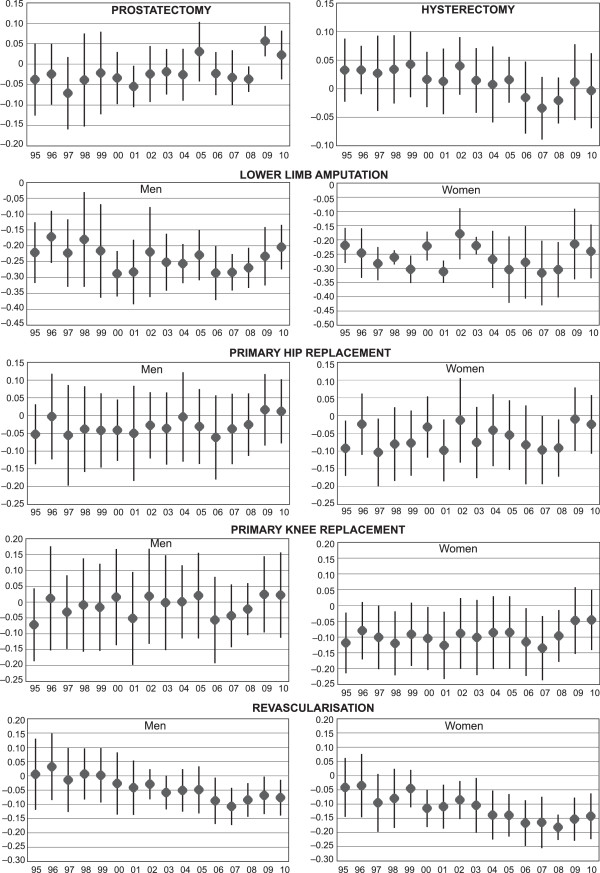
**Socioeconomic differences in common elective procedures in Finland in 1995–2010.** Concentration indices (and their 95% confidence intervals) for socioeconomic differences in common elective procedures among men and women aged 25–84 years in Finland between 1995 and 2010.

The overall rate of revascularisation operations increased rapidly among both genders from 1995 to 2005 and slowly decreased after that among both men and women (Table 2). Primary hip replacement operations increased throughout the study period among both genders. Rates for primary knee replacement operations almost tripled among both genders. Lumbar disc operation rates remained relatively stable in the 1990s and decreased in the 2000s among both men and women. Lumbar fusion operation rates increased during the study period among both genders. Hysterectomy rates decreased throughout the study period, as did prostatectomy rates. Lower limb amputation rates remained stable among men at 57 per 100,000 person years and decreased slightly among women, while appendictomy rates decreased somewhat among both genders.Figure 2 shows income group differences in elective procedures measured by concentration index during the study years. Three distinct patterns were detected in the elective procedures studied. An inverse stepwise pattern was found by income in lower limb amputation risk throughout the study period among both genders: the higher the income group, the lower the amputation rate. The concentration indices remained relatively stable throughout the study period. A similar inverse pattern was found in primary hip replacement operations among both genders and in knee replacement operations among women throughout the study period, but the patterns were, for the main part, not statistically significant. Among men, a linear trend of decreasing relative differences was found in concentration indices for hip replacement operations (p = 0.040).

**Table 2:** **Age-standardised operation rates in nine elective surgical operations in Finland in 1995 - 2010**

		Revascularisation	Primary hip replacement	Primary knee replacement	Lumbar disc operation	Lumbar fusion operation	Prostatectomy hysterectomy	Lower limb amputation	Appendectomy
		10 ^5^ 95% CI	10 ^5^ 95% CI	10 ^5^ 95% CI	10 ^5^ 95% CI	10 ^5^ 95% CI	10 ^5^ 95% CI	10 ^5^ 95% CI	10 ^5^ 95% CI
Men	1995	319 (309,329)	122 (115,128)	61 (56,66)	115 (110,120)	51 (48,55)	415 (403,427)	57 (53,62)	171 (164,177)
	2000	390 (379,400)	139 (132,145)	97 (92,103)	116 (111,121)	61 (57,65)	349 (338,359)	63 (59,68)	143 (137,149)
	2005	493 (481,504)	191 (184,198)	176 (169,183)	88 (84,92)	129 (123,135)	353 (343,363)	55 (51,59)	130 (124,135)
	2010	426 (416,436)	185 (179,192)	191 (184,197)	84 (80,88)	90 (85,94)	298 (290,307)	57 (53,61)	120 (115,125)
Women	1995	86 (81,91)	149 (143,155)	139 (134,145)	81 (77,85)	48 (45,52)	590 (579,601)	35 (32,37)	200 (193,206)
	2000	126 (120,131)	150 (144,156)	192 (185,199)	75 (71,79)	59 (55,63)	552 (541,563)	30 (27,33)	152 (146,158)
	2005	159 (153,165)	196 (189,202)	318 (310,326)	64 (61,68)	153 (148,159)	398 (389,407)	23 (21,25)	130 (124,135)
	2010	134 (129,139)	199 (193,205)	320 (312,328)	62 (59,66)	91 (87,96)	314 (306,322)	24 (22,26)	129 (123,134)

Trends in operation rates among men and women aged 25–84 years in Finland between 1995 and 2010 (Age-standardised rates per 100 000 and their 95% CI).

In revascularisation rates, a shift in the income pattern happened in the early 2000s among both genders. Among men, socioeconomic differences in operation risk were small at the beginning of the study period. A pattern of increasing operation risk by decreasing income emerged during the mid-2000s, although the operation risk ratios did not differ between the two lowest income groups. The income group pattern emerging during the study period was also statistically significant between 2005 and 2010, measured by the concentration index. Among women a similar pattern of increasing operation risk by decreasing income emerged somewhat earlier. The income group difference measured by concentration index was also statistically significant among women from 2000 onwards. The trend was also statistically significant among both men (p < 0.001) and women (p < 0.001).In primary knee replacement operations among men and prostatectomies, in lumbar disc operation or lumbar fusion operation risk ratios, and in appendectomies no systematic socioeconomic differences were detected among either men or women (no data shown). A pattern of decreasing socioeconomic differences was found in hysterectomies (Figure 2), in which the distribution of operations was pro-rich (while not statistically significant) at the beginning of the study period but as operation risk dropped during the study period, income group differences in them disappeared. The trend of decreasing differences was also statistically significant (p < 0.001).

## Discussion

The overall use of somatic specialised hospital care as well as non-surgical admissions decreased considerably from 1995 to 2010. This is likely to reflect the improvement of population health on the one hand, and the advances in health care technology and changes in treatment practices shifting focus from hospital inpatient care to ambulatory care on the other hand. The use of hospital care was inversely related to socioeconomic position among both genders throughout the study period: the lower the income, the higher the hospitalisation rate, which is concordant with earlier evidence from Finland and elsewhere [4, 5]. Our study adds to the literature by examining long-term trends in somatic specialised hospital care. However, recent studies have reported increasing income group differences in mortality suggesting that the health gap between socioeconomic groups is increasing, [18] which was only reflected in non-surgical admissions in the current results.

An interesting finding of our study was that while there were increasing socioeconomic differences in non-surgical admissions, practically no income group differences were found in surgical care. Further, while surgical admission rates decreased or remained relatively stable in other income quintiles during the study period, they increased in the two highest quintiles among men. These diverging trends may reflect increasing differences in the content of care as suggested by results from earlier research indicating that new technologies are first applied to high-income patients [4, 9, 11, 19]. Another plausible explanation would be that poorer access to ambulatory care among low-income groups [2, 3] may delay the diagnosis and result in more complications and thus decrease the possibility for surgical care.

When examining common elective surgical procedures, three distinct pictures emerged in terms of their socioeconomic distribution. Somewhat higher procedure rates in low-income groups were found in primary hip and knee replacement operations especially among women. In coronary revascularisations, however, the procedures were more common among high income earners at the beginning of the study period, in line with earlier research both from Finland [4, 6] and elsewhere [19–21]. During the study period, revascularisation rates increased rapidly and by the early 2000s, a pattern of increasing procedure rates with decreasing income emerged. The pattern found in revascularisations is in line with earlier evidence on the differences in coronary heart disease mortality [18, 22]. However, earlier research has also reported increasing coronary heart disease mortality differences between socioeconomic groups [18] and an earlier study from Finland suggests that if need is approximated with coronary heart disease incidence or coronary mortality, there are still large pro-rich income group differences in terms of access to revascularisation [23]. Hence, while the distribution of coronary revascularisations was pro-poor in our study, there is still likely to be a need for improvements in coronary care among the low-income groups.

Lower limb amputations are an example of procedures that patients eventually end up after serious vascular complications of chronic disease, especially diabetes. Our finding of stable differences in amputation rates is thus likely to reflect stable differences in emergence of complications in diabetes in particular. These results suggest a need to improve secondary preventive measures among low-income patients with increased risk of vascular complications. Our finding is in line with earlier research concerning socioeconomic differences in amputations among persons with diabetes in Finland [24] and persons with critical limb ischaemia internationally [25, 26]. No systematic socioeconomic patterns were found in lumbar disc or lumbar fusion operations, prostatectomies or appendectomies. In hysterectomies, earlier research from Finland has shown a pro-rich distribution of the procedure [4, 6]. Our results suggest that the current practice is more in line with evidence-based guidelines reflecting a requirement for caution, careful selection of patients to achieve optimum benefit and alternatives to surgical intervention [27]. The decline in overall hysterectomy rates also coincided with the publication of results from a Finnish RCT study which compared hysterectomies with levonorgestrel-releasing intrauterine devices for treating menorrhagia [28]. The study also influenced the national clinical guidelines for the treatment of excess menstrual bleeding underlining pharmaceutical treatment of menorrhagia.

A major strength of our study was that we used individual-level data on hospital use among all Finnish residents over a period of 16 years derived from the Hospital Discharge Register, the quality and coverage of which has been reported to be, in general, good. A recent systematic review reported that more than 95 per cent of discharges could be identified from the register and that the positive predictive value, i.e. the proportion of register-detected cases that are confirmed to be true-positives according to external data varied between 75% and 99% for common diagnoses [29]. We were also able to use individually linked income data derived from the registers of the tax administration and the Social Insurance Institution, allowing us to avoid ecological bias. Furthermore, we used family income instead of individual income, which is likely to produce more robust results since family income is less likely to be sensitive to reverse causation. A weakness of our study is that the Hospital Discharge Register does not collect clinical data which therefore could not be controlled for. A major weakness of our study is that we could not control for need for care and thus cannot evaluate whether care is distributed according to need. However, this is an in inherent weakness of all studies examining the total population and/or overall use of hospital care. These studies, as the current one, need to rely on indirect inference to reports of socioeconomic differences in morbidity and mortality on the population level. Earlier research concerning both mortality and morbidity reports consistent systematic socioeconomic differences [17, 18, 22] and increase in them over time [18]. These results suggest large and diverging health care needs between socioeconomic groups.

Access to health care is a complicated issue for study and use of services is often used as a proxy. There are, however, both demand side and supply side barriers that may influence the use of services. Demand side barriers including, e.g. socioeconomic factors, age, earlier experience of the services and attitudes can result in the patient not seeking the care needed. Supply side barriers are related, e.g. to the organisation and availability of services, costs and waiting times for care. Some survey studies have attempted to overcome these limitations by examining unmet need of services [30–32]. The results have mainly been condordant: unmet need is distributed pro-poor. While it is not possible to examine unmet need using register-based data, these results suggest that our results concerning differences in use of services give a rather concervative estimate of socioeconomic differences in access to care.

We used concentration indices to estimate the socioeconomic distribution of admissions and surgical procedures. The index provides comprehensive summary information about the whole distribution of the studied outcome in a single value, which is a particular advantage when making comparisons over time or between genders. When interpreting the results it should be borne in mind that the index only measures relative differences between income groups.

The changes in the socioeconomic distribution of somatic specialist care found in the current study are small considering that they have happened in the context of major changes both in the national economy and in resources within health care. Finland experienced an exceptionally deep economic recession in the early 1990s. As a result, Finland’s GDP was cut by over 10% in just a few years [33]. In 2008–2009 the slowing of the global economy dragged Finland down and the Finnish economy contracted by almost 10%. These recessions do not seem to have had an effect on the use of somatic specialised care or its distribution, since according to our results the reduction of hospitalisations has been relatively steady throughout the study period in all income groups. This is likely to be due to a simultaneous increase in the resources allocated to health services from the beginning of the 1990s mainly to specialised care and medicine costs [33]. Total health care costs have increased from 11 in 1995 to 16 billion in 2010 (in 2010 prices). There have also been increases in funding for some elective operations and in specialist doctors, especially cardiologists in Finland from the late 1990s. Earlier research into specialised care in Finland suggests that an increase in resources tends to also increase equity in the use of services [6, 34]. The current results suggest that this seems to be the case in at least some common elective operations.

Other potential explanations for the improvements in access to elective surgical operations identified above include the development and implementation of clinical guidelines, for hip and knee replacement [35] and treatment of coronary heart disease for example [36, 37]. Furthermore, some reforms have been introduced to improve access to services. In 2005, a waiting time guarantee was introduced to Finnish legislation and uniform criteria for obtaining non-emergency in-patient and out-patient care have been introduced for more than 190 diseases. These reforms may have increased equity in access to some operations while equity in access to specialist care in general may not have improved in relation to need.

## Conclusions

Between 1995 and 2010 the use of somatic specialist hospital care declined in line with improving population health. Simultaneously, relative health differentials between socioeconomic groups increased, but this was only reflected in the distribution of non-surgical care and in some elective procedures in the current study. Further research is needed to evaluate whether the use of somatic specialist hospital care, and especially surgical care, is currently distributed according to need or whether there is need to improve access to and content of care among the low-income groups in order to improve equity in health care.

## Authors’ information

KM holds a PhD in sociology and works as a research director in the National Institute for Health and Welfare (THL). A main focus of her research has been equity issues in health services research. MA holds a MSc in biostatistics and works as a statistician in THL. He has long experience in analysing register based data. IK holds a PhD in public health and works as a research professor in THL. A main focus of his research has been equity issues in health services research. He also works part time (20%) as a professor of health services research at University of Tampere, School of Health Sciences.
